# *Claroideoglomus etunicatum* and *Bacillus thuringiensis* Affect the Growth of the Invasive Plant *Ageratina adenophora* and Its Defense Against the Specialist Herbivore *Procecidochares utilis*

**DOI:** 10.3390/microorganisms12122438

**Published:** 2024-11-27

**Authors:** Ewei Du, Pengcun Li, Wenyuan Zhao, Rongchao Luo, Yaping Chen, Minghong Lu, Zhongxiang Sun, Furong Gui

**Affiliations:** 1 State Key Laboratory for Conservation and Utilization of Bioresources in Yunnan, College of Plant Protection, Yunnan Agricultural University, Kunming 650201, China; duewei123@sina.com (E.D.); lpc210367@outlook.com (P.L.); wyzhaoz@163.com (W.Z.); l13097482720@126.com (R.L.); cyp83@ynau.edu.cn (Y.C.); szx@ynau.edu.cn (Z.S.); 2 Nation Agricultural Technology Extending and Service Center, Beijing 100125, China; luminghong@agri.gov.cn; 3 Graduate School, Yunnan Agricultural University, Kunming 650201, China

**Keywords:** microbe–plant–herbivore interactions, arbuscular mycorrhizal fungi, *Bacillus*, invasive plant, defense–growth tradeoffs

## Abstract

Exotic plants can selectively recruit beneficial microorganisms, such as arbuscular mycorrhizal fungi (AMFs) and *Bacillus* spp., during their invasion process to enhance growth and competitiveness by improving nutrient absorption and strengthening defense capabilities against herbivores. However, research in the context of invasive plants remains limited. In this study, a greenhouse pot experiment was conducted to examine the effects of different treatments on the growth and defense of *Ageratina adenophora*. The treatments included no inoculation, inoculation with *Bacillus thuringiensis* (BT), inoculation with arbuscular mycorrhizal fungus (*Claroideoglomus etunicatum*, CE), dual inoculation with BT and CE (BT + CE), and the presence or absence of *Procecidochares utilis*. The results showed that both CE and BT + CE significantly enhanced nutrient concentration and promoted the growth of *A. adenophora*. The aboveground biomass increased by 35.48 and 53.38% under non-parasitism and by 68.03% and 103.72% under the parasitism of *P. utilis* for these two treatments, respectively. In comparison to the control *P. utilis*-parasitized *A. adenophora*, the BT, CE, and BT + CE treatments significantly increased protective enzyme activity, jasmonic acid concentration, and secondary metabolites. Our study indicates that the recruitment of *B. thuringiensis* in the rhizosphere of *A. adenophora* can enhance its defense ability, while *C. etunicatum* improved both growth and defense ability. The interaction effects of these two microorganisms enhances the regulation of growth and defense ability of *A. adenophora* against *P. utilis* parasitism, providing insights into the feedback effects of beneficial microorganisms on the interactions between invasive plants and biological control.

## 1. Introduction

The number of invasive plant species is increasing globally, and their impacts on biodiversity, social security, and economic losses are increasingly intensifying [1,2,3]. To mitigate the losses caused by invasive plants, it is essential to gain insights into the ecological and biological mechanisms driving their spread and dominance. In recent decades, the interaction between invasive plants and soil microorganisms has increasingly become a focus of several studies, further elucidating the mechanisms underlying the invasion of exotic plant species [4,5]. The invasion of an exotic plant can alter the composition of the soil microbial community, potentially creating positive feedback that facilitates the growth of exotic plants by enhancing microbial interactions and improving nutrient cycling [6]. Numerous studies have demonstrated that arbuscular mycorrhizal fungi (AMFs) and plant-growth-promoting rhizobacteria (PGPRs) are integral to the invasion of exotic species [7,8]. For example, exotic plants can selectively accumulate AMFs in their rhizospheres to promote their growth, as well as transfer carbon and minerals from native plants to invasive plants through mycorrhizal networks, which affects the interactions between native plants and AMFs, thereby enhancing their competitive advantage [9,10]. *Bacillus* spp., a type of PGPR, can also be selectively accumulated by invasive plants, facilitating the plants’ invasion by degrading allelochemicals and improving nutrient availability [11,12]. To date, most studies on the feedback effects of AMFs and PGPRs on invasive plants have primarily emphasized enhancements in their growth and competitive advantages over native plants. However, the feedback effects of these two microorganisms on invasive plants facing herbivory have been less studied.

A common strategy to effectively control the expansion of invasive plants involves the introduction of coevolved specialist herbivores from the original range [13]. In recent decades, there has been a growing focus on multi-trophic interactions occurring in both the aboveground (herbivores) and belowground (rhizosphere biota) regions of invasive plants [14,15]. Herbivory can affect the performance and impact of soil microbes associated with invasive plants [16,17], leading to feedback interactions that enhance the herbivory defenses of these invasive species [18]. For example, herbivory by *Agasicles hygrophila* Selman & Vogt (Chrysomelidae) mediates a stronger self-reinforcing feedback effect on the invasive plant *Alternanthera philoxeroides* (Mart.) Griseb. (Amaranthaceae) by altering soil microbial communities, in contrast to native plants, thereby facilitating the invasion of *A. philoxeroides* [19]. Beneficial soil microorganisms, particularly PGPRs and AMFs, can enhance plants’ tolerance to herbivores and promote compensatory growth by enhancing nutrient uptake [20,21], and they can also enhance resistance against herbivores by activating induced systemic resistance (ISR) [22,23]. The impact of these beneficial microorganisms on invasive plant tolerance and resistance to herbivores is influenced by their specific species [24,25].

There is increasing evidence that AMFs can induce *Bacillus* to mineralize organic phosphate in the rhizosphere by transporting carbon-rich exudates [26]. Similarly, the presence of *Bacillus* makes phosphate transfer within AMF mycelia more efficient [27]. This interaction can promote plant growth and production [28], improve soil nutrition and quality [29], and enhance the plant’s resistance to both biotic stress (nematodes, pathogens, and herbivores) and abiotic stresses (drought and salinity) [30,31,32,33]. Furthermore, the interaction between AMFs and *Bacillus* has been shown to enhance the invasive ability of exotic plants in new environments [34]. The co-inoculation of *R. intraradices*, which is the dominant AMF, and *Bacillus megaterium* de Bary (Bacillaceae), which is the dominant *Bacillus* species, within the rhizosphere of the invasive plant *Flaveria bidentis* (L.) Kuntze (Asteraceae) further promoted the plant’s growth and increased its competitive advantage over native plants compared to single inoculation [35]. Studies examining AMFs, *Bacillus* and their interactions in the context of invasive plant resistance, particularly concerning the release of coevolved specialized enemies, are needed to make progress in addressing the challenges posed by invasive plants and their biological control.

*Ageratina adenophora* (Spreng.) R. M. King & H. Rob (Asteraceae), also known as the Crofton weed, is native to Mexico and Costa Rica and has become a noxious invasive weed in approximately 40 countries, representing one of the most problematic invasive species globally [36,37]. Since its introduction to China in the 1940s, due to its rapid adaptive evolution during expansion, it has invaded different habitats in southwestern and central China, causing huge ecological and economic losses [38,39,40]. *Procecidochares utilis* Stone (Diptera: Tephritidae), a specialist herbivore of *A. adenophora*, can induce galls in the damaged parts of the plant and obstruct its nutrient channels, thereby inhibiting its growth and reproduction [41,42]. Previous studies have revealed that AMFs and *Bacillus* spp. play vital roles in the invasion of *A. adenophora* [43,44]. Specifically, *Septoglomus constrictum* (Trappe) Sieverd., G. A. Silva & Oehl (Glomeraceae), a dominant AMF, facilitates nutrient absorption through its extensive hyphal network, and *Bacillus cereus* Frankland & Frankland (Bacillaceae), a dominant *Bacillus* spp., increases nutrient availability in the soil; both species are found in the rhizosphere of *A. adenophora*. Moreover, the cooperation between *S. constrictum* and *B. cereus* enhances the growth and competitive advantage of *A. adenophora* [45]. Because soil microorganisms can enhance plant defense mechanisms, it is essential to further investigate the interaction between invasive plants and various herbivores, as well as the relationship between rhizosphere microorganisms. Previous studies have also found that the dominant AMF *Claroideoglomus etunicatum* (W. N. Becker & Gerd.) C. Walker & A. Schüßler and the dominant *Bacillus* spp. *Bacillus thuringiensis* Berliner (Bacilllaceae) in the rhizosphere of *A. adenophora* enhanced plants*’* defense against the generalist *Aphis gossypii* Glover (Aphididae) in an invaded area by improving the plant’s chemical defenses [46]. Parasitism by *P. utilis* increased the density of *B. cereus* in the rhizosphere of *A. adenophora*, but *B. cereus* did not positively affect the parasitism of *P. utilis*, possibly because the indirect effect of *B. cereus* on *P. utilis* through the plant was not significant [47]. However, the role of *C. etunicatum* and *B. thuringiensis* and their interaction in facilitating the tolerance of and resistance to the specialist herbivore *P. utilis* in *A. adenophora* remain unclear.

This study aimed to investigate the dominant AMF and *Bacillus* in the rhizosphere of *A. adenophora*, as well as their interactions within the context of the relationship between *A. adenophora* and its specialist herbivore. We hypothesized that AMFs, *Bacillus*, and their interactions present in the rhizosphere soil of *A. adenophora* would exert different effects on the plant’s growth and defense ability. To test this hypothesis, we measured the growth and defense parameters of *A. adenophora* following inoculation and co-inoculation treatments, as well as in the absence or presence of parasitism by *P. utilis*. We also assessed the AM fungal colonization rate and *Bacillus* density in *A. adenophora* across the different treatments and analyzed their correlations with growth and defense ability. Finally, we evaluated the durations of the development of *P. utilis* across the different treatments.

## 2. Materials and Methods

### 2.1. Soil, Plants, and Insect Preparation

Soil was collected from a depth of 20–40 cm at Yunnan Agricultural University (Kunming, China; 25°08′30″ N, 102°45′13″ E; elevation: 1940 m). Vermiculite was purchased from Dounan Plant and Flower Co., Ltd. (Kunming, China). The growing medium consisted an equal volume mixture of soil and vermiculite ([(Mg,Fe,Al)_3_ [Si,Al]_4_O_10_(OH)_2_·4H_2_O], Dounan Plant and Flower Co., Ltd., Kunming, China) and had a pH of 6.25 (measured in water, 1:5 *w*/*v*). There was 15.502 g kg^−1^ of organic matter, and nutrient concentrations (g kg^−1^) were 0.899 for total nitrogen, 0.351 for total phosphorus, and 40.03 for total potassium. The available nitrogen, phosphorus, and potassium levels were determined to be 20.28 μg/g, 5.089 μg/g, and 32.32 mg/kg, respectively. The mixtures were sterilized by autoclaving them at 121 °C for 2 h, after which 1 kg of the sterilized soil was placed in plastic pots (20 × 13 × 14 cm; length × width × height) prior to planting. The pots had been soaked in 75% ethanol for 10 min and were allowed to dry before use.

Seeds of the invasive plant *A. adenophora* were obtained from Yunnan Agricultural University. Then, they were surface-disinfected for 5 min in 5% sodium hypochlorite and, subsequently, in 75% ethanol for 1 min, before being rinsed five times with sterile tap water.

*Procecidochares utilis* galls with *A. adenophora* were collected randomly from the area near the Kunming suburb and brought to the greenhouse (average temperature: 25.6 ± 1.5 °C; average humidity: 77.5%) for planting. When adults of *P. utilis* emerged from the galls, we placed them in a cage containing potted *A. adenophora* for continued cultivation. This process was repeated until a stable population was established, and the adults of *P. utilis* were used for the experiment.

### 2.2. Claroideoglomus etunicatum and Bacillus thuringiensis

*Claroideoglomus etunicatum* and *B. thuringiensis* were selected as representative species of AMFs and *Bacillus*, respectively, to test their effects on the response of *A. adenophora* to the specialist herbivore *P. utilis*. The spores of *C. etunicatum* were isolated from the rhizosphere soil of *A. adenophora* using the wet sieving and sucrose centrifugation method, followed by scanning electron microscopy [48,49]. *Sorghum vulgare* Pers. (Poaceae) and *Trifolium repens* L. (Fabaceae) were used as the host plants, and a sand/soil mixture (1:1, *v*/*v*) was used as the culture medium in a sterile culture experiment for four months. Then, the inocula were air-dried and sieved (2 mm) to obtain a mixture of rhizospheric soil containing fine root fragments, hyphae and spores. At harvest, the spore density was determined to be 40 spores g^−1^ of inoculum. The *B. thuringiensis* strain A47 (GenBank accession: OM149780) was isolated from the rhizosphere soil of *A. adenophora* cultivated in Kunming, China. A 100 μL aliquot of the preserved strain suspension was cultured on nutrient agar plates at 30 °C for 12 h, after which single colonies were selected and transferred to a nutrient liquid medium in which they were incubated under 180 rpm rotation at 28 °C for 20 h. The suspensions of *B. thuringiensis* were then diluted and adjusted to a concentration of 10^8^ CFU/mL.

### 2.3. Experimental Design

The experiment was a factorial experiment with a completely randomized design and two factors (inoculants × parasitism treatments, Figure S1). Four inoculant treatments (C: uninoculated treatment; CE: *C. etunicatum* alone; BT: *B. thuringiensis* alone; and CE + BT: co-inoculation with *C. etunicatum* and *B. thuringiensis*) with two parasitism treatments (parasitism by *P. utilis* and no parasitism) were carried out. In the *C. etunicatum* inoculation treatment pots, 100 g of AMF inoculum was added 1 cm below the soil surface. The non-AMF pots received 100 g of autoclaved *C. etunicatum* inoculum along with the filtrate (<20 mm) of the AMF inoculum to maintain consistent nutrient content. For the *Bacillus* inoculation treatments, 10 mL of *B. thuringiensis* bacterial suspension (10^8^ CFU/mL) was added to each pot, while the non-*Bacillus* pots received 10 mL of sterilized bacterial suspension. Five seeds were sown in pots, and two weeks after germination, excess plants were removed, leaving one plant of the same size in each pot, with five replicates for each treatment. The plants were watered with sterile water every two days and supplemented with 100 mL of Hoagland solution every two weeks. After germination, each pot was thinned to contain only one plant. The pots were cultivated in a controlled greenhouse maintained at 25 °C with a 10 h L/14 h D cycle. After three months of growth, each *A. adenophora* plant was covered with a 100-mesh cage (45 cm length, 30 cm width, and 60 cm height). No *P. utilis* was placed in the cages of seedlings in the no-parasitism treatments. In the parasitism treatments, one pair of newly emerged *P. utilis* adults was placed in each cage. Data were recorded for the initiation of duration of *P. utilis* development, the number of galls, and the emergence of adult *P. utilis* from each gall.

### 2.4. Measurement

#### 2.4.1. Colonization of AMF and Density of *Bacillus*

After assessing the growth and development duration of *P. utilis* parasitizing on *A. adenophora*, fresh leaves were collected for testing defense indicators, and then the plants and rhizosphere soil were harvested. The roots were washed to remove soil, and 0.2 g of roots from each treatment was used to measure the mycorrhizal colonization rate. The roots from each sample were cut into 2 cm pieces. Subsequently, they were rinsed in 10% KOH, acidified with 2% HCl, and stained with a 0.1% acidic fuchsin solution [50]. The percentage of AMF colonization was estimated using a modified method based on McGongle et al. [51]. A total of 200 root segments were analyzed using a compound microscope (Olympus BX43, Tokyo, Japan) at 40× magnification, with five replicates per treatment. The AM fungal structures (hyphae, vesicles, arbuscules, and spores of each root sample) were scored. The colonization rate of *C. etunicatum* was calculated by combining the scores from the 200 root segments.

To explore the impacts of different treatments on the growth of *B. thuringiensis*, the density of *B. thuringiensis* in the rhizosphere was determined. A suspension dilution assay was used to estimate the density of *B. thuringiensis* [34]. Specifically, 1 g of rhizosphere soil was mixed with 9 mL of distilled water and homogenized at 200 rpm for 24 h, followed by heating at 80 °C for 10 min. After allowing the mixture to stand for 12 h, the supernatant was serially diluted, Subsequently, 0.1 mL of 10^−3^ supernatant was transferred to nutrient agar plates and incubated for 16 h at 37 °C. The results were determined as colony-forming units per gram of dry weight of soil (CFU/g) based on the volume dilution.

#### 2.4.2. Biomass and Root Growth Characteristics

The roots were then scanned using a root scanner (Epson Expression 10000XL; Epson, Long Beach, CA, USA). WinRhizo Software (WinRHIZO 2019v, Regent Instruments Inc., Québec City, QC, Canada) was used to analyze the root growth characteristics. After measuring the root characteristics, the roots were collected, both the aboveground parts and roots were oven-dried at 80 °C for 72 h, and the dry biomass of both was measured.

#### 2.4.3. Nutrient Concentrations

The concentrations of nutrients, including starch, soluble sugar, protein, total chlorophyll, carbon, nitrogen, and phosphorous, were measured. A sample of 0.2 g of dry tissue was homogenized in 80% ethanol and placed in a water bath at 80 °C for 30 min. The concentrations of total sugars and starch were estimated colorimetrically using the phenol–sulfuric acid method described by Dubois et al. [52], while the reducing sugars were quantified using the Nelson–Somogyi method described by Oser [53]. The soluble protein concentration was determined using the Bradford method [54]. The total chlorophyll concentration in the plants was determined using the spectrophotometric method of Lichtenthaler [55]. Following drying, the plant material was ground into a powder (Tissuelyser-48, Shanghai Jingxin Industrial Development Co., Ltd., Shanghai, China), and 0.2 g of tissue was used to analyze the concentrations of carbon (C), nitrogen (N), and phosphorus (P). The C concentration was measured using an NC analyzer (Thermo Fisher Scientific, Waltham, MA, USA), the N concentration was measured using the micro-Kjeldahl method [56], and the P concentration was measured using inductively coupled plasma spectroscopy [57].

#### 2.4.4. Antioxidant Enzyme Activities and Defense Hormones

A phytochemical analysis was conducted to the evaluate the effects of different inoculants and parasitism by *P. utilis* on the activities of antioxidant enzymes and the concentrations of defense hormones in plant leaves. Approximately four leaves surrounding the galls were collected for each treatment. Then, 0.2 of leaves were weighted; the antioxidant enzyme (superoxide dismutase (SOD), polyphenol oxidase (POD), phenylalanineammonialyase (PAL), and polyphenol oxidase (PPO)) were extracted; and the activity of those enzymes was analyzed using the assay kits (A001-1-2 for SOD, A084-3-1 for POD, A137-1-1 for PAL, and A136-1-1 for PPO; Nanjing Jiangcheng Bioengineering Institute, Nanjing, China), in accordance with the manufacturer’s instructions. A total of 0.2 g of leaves was weighed, followed by the addition of 1 mL of methanol for thorough grinding, and then 9 mL of phosphate buffer was added for homogenization. Following centrifugation at 4000 rpm for 10 min, the concentrations of jasmonic acid (JA) and salicylic acid (SA) were quantified using ELISA kits (Jiangsu Boshen Biotechnology Co., Ltd., Nanjing, China), following the manufacturer’s protocol. The optical density (OD) values of each reaction mixture were measured using a microplate reader (Varioskan LUX, Thermofisher, USA).

#### 2.4.5. Secondary Metabolites Concentrations

A 0.1 g mass of dry tissue was extracted using 60% ethanol, followed by three 20 min ultrasound sonication treatments. The mixture was then centrifuged at 10,000 rpm for 5 min at 4 °C. The Prussian blue assay was used to estimate the total phenol concentration [58]. The total flavonoid concentration was estimated using the Al(NO_3_)_3_ colorimetric method [59]. The tannin concentration was measured with a spectrophotometer (OD = 760 nm) using the Folin–Na_2_CO_3_ colorimetric method [60,61].

#### 2.4.6. Duration of Development of *Procecidochares utilis*

We measured the duration of the development of *P. utilis* as described by Gao et al. [41] across the different treatments in this experiment. Briefly, it was divided into four stages: (a) the period from the beginning to when the diameter of the gall exceeded 4 mm was called the “galls visible” period; (b) the period from the start to when a thin epidermal layer had formed at the outer end of the exit tunnel and become visible as a “window” on the surface of the gall was called the “window visible” period; (c) the period from the initiation time to the emergence of the first adult was called the “adult emergence” period; and (d) the period from adult emergence to death was called the “lifespan of adults”. When the galls on the *A. adenophora* had reached the “window visible” stage, each gall was covered with a mesh bag, and the number of adult emergences from each gall in each plant was recorded.

### 2.5. Statistical Analysis

The effects of the inoculum and *P. utilis* parasitism on the plant growth parameters were tested using two-way analyses of variance (ANOVAs) following the confirmation of a normal distribution. A one-way ANOVA was performed to investigate the differences in AMF colonization and *Bacillus* density across the different inoculum treatments, as well as to evaluate the impact of the inoculum on the duration of the development of *P. utilis*. Multiple comparisons among the groups were conducted using the least significant difference (LSD) method. To elucidate the patterns of *A. adenophora’s* responses to different microbial inoculations and parasitism by *P. utilis*, principal component analysis (PCA) was conducted using data related to biomass, nutrient content, root growth characteristics, antioxidant enzyme activities, secondary metabolites, and defensive hormone concentrations. Additionally, the regression analysis between AMF colonization or *Bacillus* density and the duration of the development of *P. utilis* was calculated. All the analyses were performed using SPSS 16.0 (SPSS, Chicago, IL, USA). Effects were considered significant if *p* < 0.05.

## 3. Results

### 3.1. Effects of Inoculum and Parasitism by Procecidochares utilis on AMF Colonization and Bacillus Density of Ageratina adenophora

**Figure 1:**
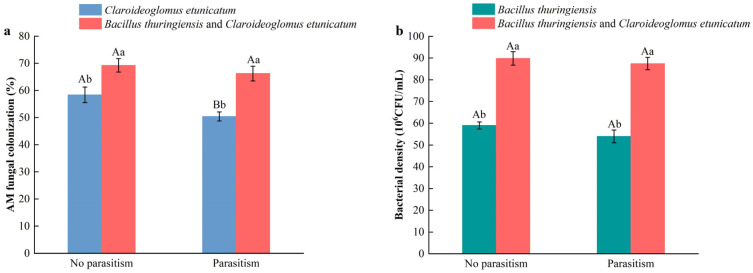
Effects of different inoculant treatments and *Procecidochares utilis* parasitism on the AMF colonization (**a**) and bacterial density (**b**) of *Ageratina adenophora*. CE, inoculated with *Claroideoglomus etunicatum*; BT, inoculated with *Bacillus thuringiensis*; BT + CE, co-inoculated with *B. thuringiensis* and *C. etunicatum*. Different lowercase letters on the bars represent significant differences between the inoculation treatments (LSD test, *p* < 0.05). Different uppercase letters on the bars represent significant differences between parasitism by *Procecidochares utilis* and no parasitism (*t*-test, *p* < 0.05). Data are means ± SE (*n* = 5).

No arbuscular mycorrhizal fungal colonization was found following treatments without AM fungal inoculation either with or without *P. utilis*. Following the inoculation with CE treatment, the AM fungal colonization rate of *A. adenophora* was 58.83% and 50.39% with and without *P. utilis* parasitism, respectively (Figure 1a). Following the dual inoculation with BT + CE treatment, the AM fungal colonization rate of *A. adenophora* was 69.22% and 66.21% with and without *P. utilis* parasitism, respectively. The results showed that parasitism by *P. utilis* significantly decreased the AM fungal colonization rate of *A. adenophora* under *C. etunicatum* inoculation treatment. Conversely, dual inoculation with *B. thuringiensis* and *C. etunicatum* significantly increased the AM fungal colonization rate of *A. adenophora* both with and without *P. utilis* parasitism.

No bacterial colonies were found following treatments without *Bacillus* inoculation either with or without *P. utilis*. Following the inoculation with BT treatment, the density of *B. thuringiensis* in the rhizosphere soil of *A. adenophora* was 59 × 10^6^ and 54 × 10^6^ CFU/mL with and without *P. utilis* parasitism, respectively (Figure 1b). Following the dual inoculation with BT + CE treatment, the density of *B. thuringiensis* in the rhizosphere soil of *A. adenophora* was 89.9 × 10^6^ and 87.4 × 10^6^ CFU/mL with and without *P. utilis* parasitism, respectively. The results showed that parasitism by *P. utilis* had no effect on the density of *Bacillus* in *A. adenophora’s* rhizosphere under inoculation with *B. thuringiensis* or dual inoculation with *B. thuringiensis* and *C. etunicatum*. The dual inoculation with *B. thuringiensis* and *C. etunicatum* significantly increased the density of *B. thuringiensis* in the rhizosphere soil of *A. adenophorain* both with and without *P. utilis p*arasitism.

### 3.2. Effect of Inocula and Parasitism by Procecidochares utilis on the Growth of Ageratina adenophora

**Figure 2:**
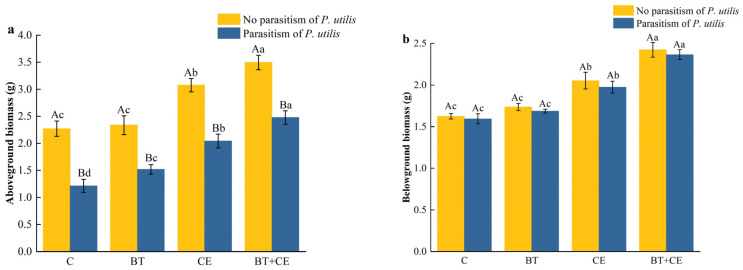
Effects of different inoculant treatments and *Procecidochares utilis* parasitism on the aboveground (**a**) and belowground (**b**) biomass of *Ageratina adenophora*. C, control; BT, inoculated with *Bacillus thuringiensis*; CE, inoculated with *Claroideoglomus etunicatum*; BT + CE, co-inoculated with *B. thuringiensis* and *C. etunicatum*. Bars with different lowercase letters represent significant differences between the inoculation treatments by LSD test (*p* < 0.05). Bars with different uppercase letters represent significant differences between parasitism by *Procecidochares utilis* and no parasitism by *t*-test (*p* < 0.05). Data are means ± SE (*n* = 5).

The aboveground biomass of *A. adenophora* was affected by the inoculant treatment (*F* _(3,32)_ = 137.71, *p* < 0.001), parasitism by *P. utilis* (*F* _(1,32)_ = 387.31, *p* < 0.001), and the interaction of inoculation and parasitism treatment (*F* _(3,32)_ = 14.95, *p* < 0.001), while its belowground biomass was only affected by the inoculant treatment (belowground biomass: *F* _(3,32)_ = 400.15, *p* < 0.001; Table S1). Specifically, the aboveground biomass was decreased by parasitism by *P. utilis* (Figure 2a), while the belowground biomass was not significantly affected (Figure 2b). Different inoculation treatments had different effects on the aboveground and belowground biomass of *A. adenophora* (Figure 2a,b). In the no-*P. utilis* parasitism treatments, inoculation with *B. thuringiensis* had no significant effect on the aboveground and belowground biomass of *A. adenophora* compared with the uninoculated treatment, while inoculation with *C. etunicatum* and co-inoculation with *B. thuringiensis* and *C. etunicatum* significantly increased the aboveground and belowground biomass of *A. adenophora* (*p* < 0.01). The aboveground and belowground biomass of *A. adenophora* increased by 35.45% and 26.43% upon inoculation with *C. etunicatum* and increased by 53.85% and 49.38% upon inoculation with *B. thuringiensis* and *C. etunicatum*. All three inoculation treatments in the presence of *P. utilis* parasitism significantly increased the aboveground biomass of *A. adenophora* (*p* < 0.001) by 24.85%, 68.04%, and 103.72%, respectively. Inoculation with *C. etunicatum* and co-inoculation with *B. thuringiensis* and *C. etunicatum* significantly increased the belowground biomass of *A. adenophora* (*p* < 0.001) by 23.96% and 50.35%, respectively.

The root growth characteristics of *A. adenophora* were only affected by the inoculant treatment (root length: *F* _(3,32)_ = 151.85, *p* < 0.001; root surface area: *F* _(3,32)_ = 1008.03, *p* < 0.001; root diameter: *F* _(3,32)_ = 769.85, *p* < 0.001; root volume: *F* _(3,32)_ = 291.46, *p* < 0.001; Table S1) and were not affected by the parasitism treatment or the interaction between the inoculation and parasitism treatment (Table S1). The root growth characteristics were not significantly affected by the parasitism by *P. utilis* (Table 1). Unlike inoculation with *B. thuringiensis* alone, inoculation with *C. etunicatum* and co-inoculation with *B. thuringiensis* and *C. etunicatum* significantly increased the root growth characteristics of *A. adenophora* (*p* < 0.01), and the co-inoculation had a significantly greater effect than inoculation with *C. etunicatum* alone (*p* < 0.01, Table 1). Inoculation with *B. thuringiensis* did not significantly affect the root growth characteristics in the no-parasitism treatment compared to the uninoculated treatment, but it significantly increased the root growth characteristics in the parasitism treatment.

The soluble sugar, soluble protein, and starch concentrations of *A. adenophora* were affected by the inoculant treatment (soluble sugar: *F* _(3,32)_ = 102.97, *p* < 0.001; soluble protein: *F* _(3,32)_ = 339.62, *p* < 0.001; starch: *F* _(3,32)_ = 86.97, *p* < 0.001); parasitism by *P. utilis* (soluble sugar: *F* _(1,32)_ = 805.67, *p* < 0.001; soluble protein: *F* _(1,32)_ = 2870.63, *p* < 0.001; starch: *F* _(1,32)_ = 661.76, *p* < 0.001); and the interaction between the inoculant and *P. utilis* parasitism (soluble sugar: *F* _(3,32)_ = 11.44, *p* < 0.001; soluble protein: *F* _(3,32)_ = 59.93, *p* < 0.001; starch: *F* _(3,32)_ = 23.85, *p* < 0.001, Table S1). The concentrations of soluble sugar, soluble protein, and starch in *A. adenophora* were decreased by parasitism by *P. utilis* (Table 2). The effects of different inoculants on the concentrations of soluble sugar, soluble protein, and starch in *A. adenophora* were different under parasitism by *P. utilis* and no parasitism (Table 2). In the absence of parasitism, inoculation with *B. thuringiensis* had no effect on the soluble sugar, soluble protein, and starch concentrations of *A. adenophora* compared with the control treatment, while inoculation with *C. etunicatum* and co-inoculation with *B. thuringiensis* and *C. etunicatum* significantly increased these concentrations (*p* < 0.001). In the presence of parasitism by *P. utilis*, the concentrations of soluble sugar, soluble protein, and starch were significantly increased by the inoculation treatments (*p* < 0.001).

**Figure 3:**
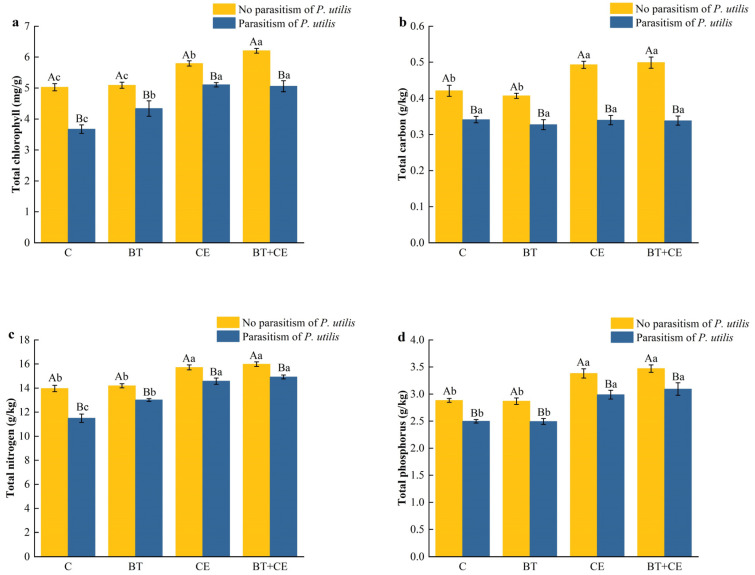
Effects of different inoculant treatments and *Procecidochares utilis* parasitism on the total chlorophyll (**a**), carbon (**b**), nitrogen (**c**), and phosphorus (**d**) concentrations of *Ageratina adenophora*. C, control; BT, inoculated with *Bacillus thuringiensis*; CE, inoculated with *Claroideoglomus etunicatum*; BT + CE, inoculated with *B. thuringiensis* and *C. etunicatum*. Bars with different lowercase letters represent significant differences between the inoculation treatments (LSD test, *p* < 0.05). Bars with different uppercase letters represent significant differences between parasitism by *Procecidochares utilis* and no parasitism (*t*-test, *p* < 0.05). Data are means ± SE (*n* = 5).

The total chlorophyll, carbon, nitrogen, and phosphorus concentrations of *A. adenophora* were affected by the inoculant treatment (chlorophyll: *F* _(3,32)_ = 225.25, *p* < 0.001; carbon: *F* _(3,32)_ = 45.28, *p* < 0.001; nitrogen: *F* _(3,32)_ = 245.49, *p* < 0.001; phosphorus: *F* _(3,32)_ = 137.43, *p* < 0.001); parasitism by *P. utilis* (chlorophyll: *F* _(1,32)_ = 868.74, *p* < 0.001; carbon: *F* _(1,32)_ = 1055.74, *p* < 0.001; nitrogen: *F* _(1,32)_ = 393.29, *p* < 0.001; phosphorus: *F* _(1,32)_ = 285.38, *p* < 0.001); and the interaction between the inoculant and *P. utilis* parasitism (chlorophyll: *F* _(1,32)_ = 22.85, *p* < 0.001; carbon: *F* _(1,32)_ = 44.14, *p* < 0.001; nitrogen: *F* _(1,32)_ = 35.42, *p* < 0.001; phosphorus: *F* _(1,32)_ = 26.04, *p* < 0.001; Table S1). Parasitism by *P. utilis* significantly decreased the chlorophyll, carbon, nitrogen, and phosphorus concentrations of *A. adenophora* (Figure 3a–d). The total chlorophyll, carbon, nitrogen, and phosphorus concentrations of *A. adenophora* were also affected by different inoculation treatments (Figure 3a–d). In the absence of parasitism, inoculation with *B. thuringiensis* had no significant effects on the concentrations of total chlorophyll, carbon, nitrogen, and phosphorus, while inoculation with *C. etunicatum* and co-inoculation with *B. thuringiensis* and *C. etunicatum* increased these concentrations (*p* < 0.001). In the presence of parasitism by *P. utilis*, the concentrations of total chlorophyll, nitrogen, and phosphorus were significantly increased by the inoculation treatments (*p* < 0.001).

### 3.3. Effect of Inocula and Parasitism by Procecidochares utilis on the Defense of Ageratina adenophora

**Figure 4:**
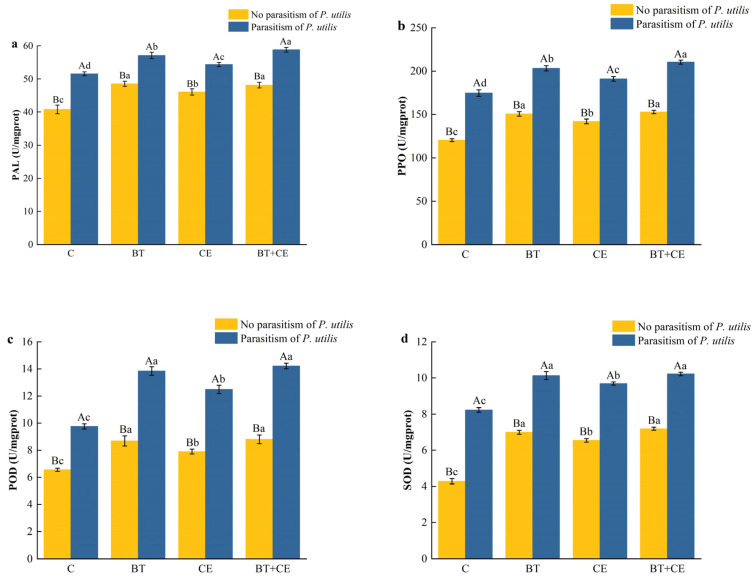
Phenylalanine ammonialyase (PAL, **a**), polyphenol oxidase (PPO, **b**), peroxidase (POD, **c**), and superoxide dismutase (SOD, **d**) activities of *Ageratina adenophora* under different inoculant treatments. C, control; BT, inoculated with *Bacillus thuringiensis*; CE, inoculated with *Claroideoglomus etunicatum*; BT + CE, inoculated with *B. thuringiensis* and *C. etunicatum*. Bars with different lowercase letters represent significant differences between the inoculation treatments (LSD test, *p* < 0.05). Bars with different uppercase letters represent significant differences between parasitism by *Procecidochares utilis* and no parasitism (*t*-test, *p* < 0.05). Data are means ± SE (*n* = 5).

The activity of four antioxidant enzymes in *A. adenophora* was significantly affected by the inoculant treatment (PAL: *F* _(3,32)_ = 164.89, *p* < 0.001; PPO: *F* _(3,32)_ = 337.82, *p* < 0.001; POD: *F* _(3,32)_ = 329.57, *p* < 0.001; SOD: *F* _(3,32)_ = 782.14, *p* < 0.001); parasitism by *P. utilis* (PAL: *F* _(1,32)_ = 1378.61, *p* < 0.001; PPO: *F* _(1,32)_ = 4226.38, *p* < 0.001; POD: *F* _(1,32)_ = 2966.51, *p* < 0.001; SOD: *F* _(1,32)_ = 6729.91, *p* < 0.001); and the interaction between the inoculant and *P. utilis* parasitism (PAL: *F* _(3,32)_ = 6.93, *p* = 0.001; PPO: *F* _(3,32)_ = 4.55, *p* = 0.009; POD: *F* _(3,32)_ = 34.29, *p* < 0.001; SOD: *F* _(3,32)_ = 27.59, *p* < 0.001). Parasitism by *P. utilis* significantly increased the antioxidant enzyme activity of *A. adenophorea*, and the inoculation treatment also significantly increased the antioxidant enzyme activity of both parasitized and non-parasitized *A. adenophora* (Figure 4a–d). Compared with the control, the PAL activity of *A. adenophora* increased by 19.01%, 12.97%, and 18.02%; the PPO activity increased by 20.10%, 18.01%, and 26.95%; the POD activity significantly increased by 32.53%, 20.43%, and 34.34%; and the SOD activity significantly increased by 63.35%, 52.98%, and 67.86% (Figure 4a–d). Upon the parasitic treatment of *P. utilis*, the PAL activity of *A. adenophora* increased by 10.69%, 5.36%, and 14.09%; the PPO activity increased by 16.29%, 9.41%, and 20.37%; the POD activity increased by 41.91%, 28.07%, and 45.72%; and the SOD activity increased by 23.07%, 17.79%, and 24.19% (Figure 4a–d).

**Figure 5:**
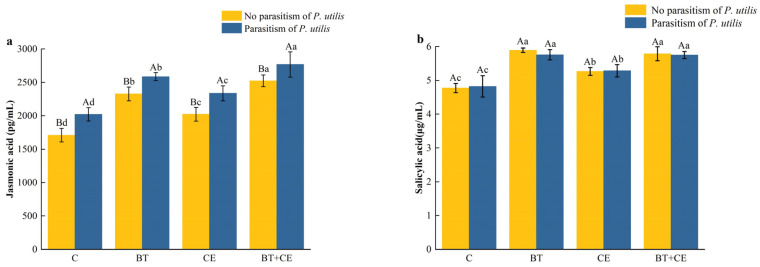
Jasmonic acid (**a**) and salicylic acid (**b**) concentrations of *Ageratina adenophora* under different inoculant treatments. C, control; BT, inoculated with *Bacillus thuringiensis*; CE, inoculated with *Claroideoglomus etunicatum*; BT + CE, inoculated with *B. thuringiensis* and *C. etunicatum*. Bars with different lowercase letters represent significant differences between the inoculation treatments (LSD test, *p* < 0.05). Bars with different uppercase letters represent significant differences between parasitism by *Procecidochares utilis* and no parasitism (*t*-test, *p* < 0.05). Data are means ± SE (*n* = 5).

The concentration of jasmonic acid in *A. adenophora* was affected by the inoculant treatment (*F* _(3,32)_ = 118.22, *p* < 0.001), parasitism by *P. utilis* (*F* _(1,32)_ = 580.41, *p* < 0.001), and the interaction between the inoculant and *P. utilis* parasitism (*F* _(3,32)_ = 11.89, *p* < 0.001; Table S1). The concentration of salicylic acid in *A. adenophora* was only affected by the inoculant treatment (*F* _(3,32)_ = 76.79, *p* < 0.001; Table S1). Parasitism by *P. utilis* increased the jasmonic acid concentration of *A. adenophora* but had no significant effect on the salicylic acid concentration (Figure 5a,b). The inoculation treatments increased the jasmonic acid and salicylic acid concentrations of *A. adenophora* in both the absence and presence of parasitism. The treatments ranked as follows in terms of how significantly they increased the jasmonic acid concentration: BT + CE > BT > CE > C (Figure 5a). They ranked as follows in terms of how significantly they increased the salicylic acid concentration: BT + CE = BT > CE > C (Figure 5b).

**Figure 6:**
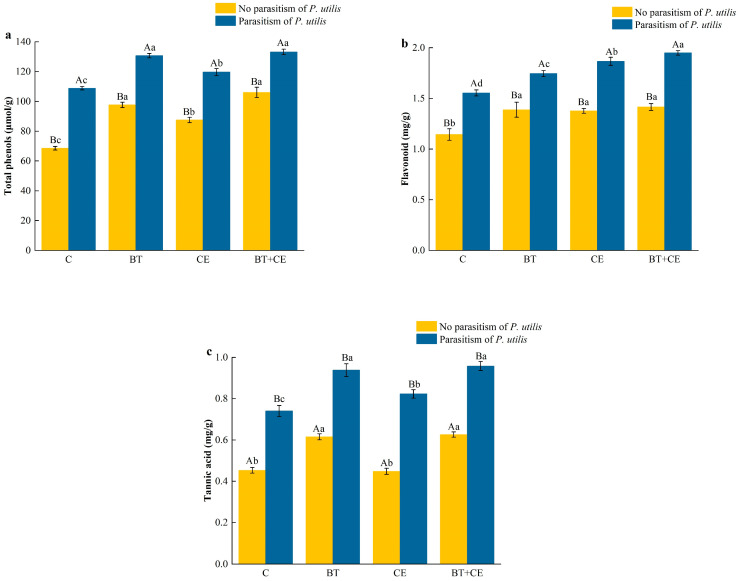
Total phenol (**a**), flavonoid (**b**), and tannic acid (**c**) contents of *Ageratina adenophora* under different inoculant treatments. C, control; BT, inoculated with *Bacillus thuringiensis*; CE, inoculated with *Claroideoglomus etunicatum*; BT + CE, inoculated with *B. thuringiensis* and *C. etunicatum*. Bars with different lowercase letters represent significant differences between the inoculation treatments (LSD test, *p* < 0.05). Bars with different uppercase letters represent significant differences between parasitism by *Procecidochares utilis* and no parasitism (*t*-test, *p* < 0.05). Data are means ± SE (*n* = 5).

The secondary metabolite concentration of *A. adenophora* was significantly affected by the inoculant treatment (total phenols: *F* _(3,32)_ = 457.22, *p* < 0.001; flavonoids: *F* _(3,32)_ = 118.13, *p* < 0.001; tannic acid: *F* _(3,32)_ = 232.09, *p* < 0.001); parasitism by *P. utilis* (total phenols: *F* _(1,32)_ = 2715.19, *p* < 0.001; flavonoids: *F* _(1,32)_ = 1125.85, *p* < 0.001; tannic acid: *F* _(1,32)_ = 2600.01, *p* < 0.001); and the interaction between the inoculant and *P. utilis* parasitism (total phenols: *F* _(3,32)_ = 18.11, *p* < 0.001; flavonoids: *F* _(3,32)_ = 8.73, *p* < 0.001; tannic acid: *F* _(3,32)_ = 7.85, *p* < 0.001; Table S1). Parasitism by *P. utilis* significantly increased the concentrations of total phenols, flavonoids, and tannic acid (Figure 6a–c). The inoculation treatment significantly increased the concentrations of secondary metabolites in *A. adenophora* in both the presence and absence of parasitism. Compared with the no-inoculation treatment in the absence of parasitism, the three inoculation treatments (BT, CE, and BT + CE) increased the total phenol concentrations by 42.39%, 27.65%, and 54.62% and the flavonoid concentration by 21.33%, 20.37%, and 23.69%, respectively (Figure 6a,b). Except for the CE treatment, the inoculation treatments increased the tannic acid concentration by 35.76% and 29.28% (Figure 6c). In the presence of parasitism, those same inoculation treatments increased the total phenol concentration by 20.06%, 9.93%, and 22.34%; the flavonoid concentration by 12.29%, 20.08%, and 25.42%; and the tannic acid concentration by 26.76%, 11.22%, and 29.49%, respectively (Figure 6a–c).

### 3.4. Development Time of Procecidochares utilis Reared on Ageratina adenophora with Inocula

**Figure 7:**
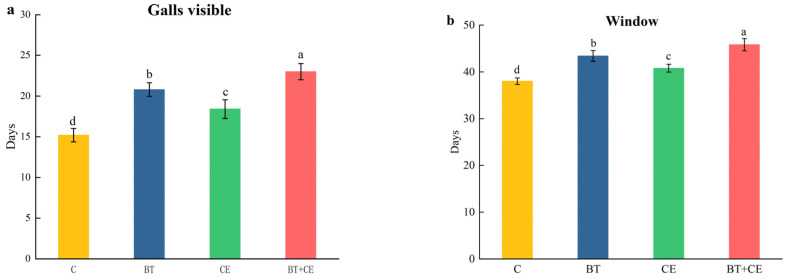

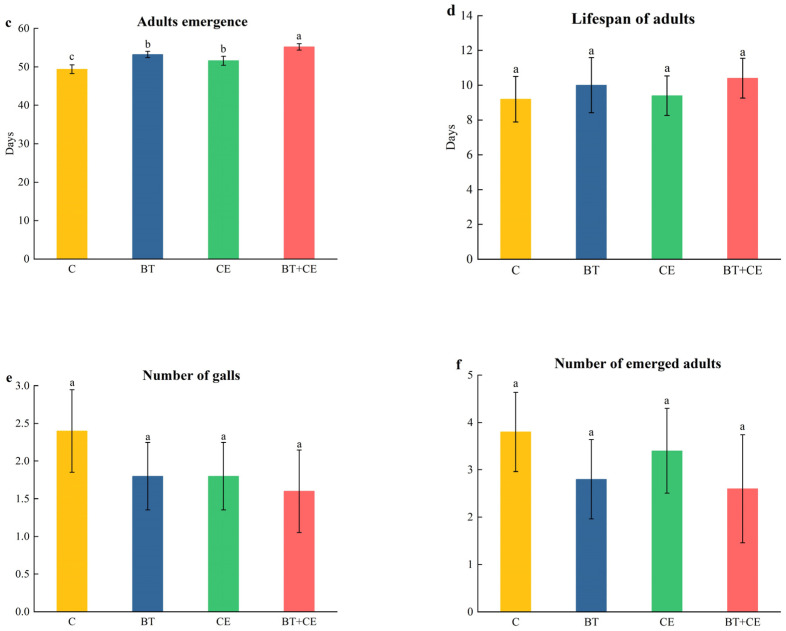
The life history parameters of *Procecidochares utilis* parasitized on *Ageratina adenophora* under different inoculant treatments ((**a**), “galls visible” duration; (**b**), “window” duration; (**c**), “adult emergence” duration; (**d**), adult lifespan; (**e**), number of galls; (**f**), number of emerged adults). C, control; BT, inoculated with *Bacillus thuringiensis*; CE, inoculated with *Claroideoglomus etunicatum*; BT + CE, inoculated with *B. thuringiensis* and *C. etunicatum*. Different letters on the bar show significant differences between the inoculated treatments by LSD test (*p* < 0.05). Data are the means ± SE.

Our study found that the rhizosphere microbes of *A. adenophora* could significantly prolong the duration of the development of *P. utilis* reared on *A. adenophora* (Figure 7a–d). In *P. utilis*-parasitized *A. adenophora*, inoculation with *B. thuringiensis* prolonged the “galls visible” duration, “window” duration, “adult emergence” duration, and adult lifespan by 36.84%, 14.21%, 7.69% and 8.70%, respectively; inoculation with *C. etunicatum* prolonged these by 21.05%, 7.37%, 4.45% and 2.17%, respectively; and co-inoculation with both *B. thuringiensis* and *C. etunicatum* prolonged these by 51.32%, 20.53%, 11.74% and 11.54%, respectively. In contrast to the effects on the duration of development, inoculation with *B. thuringiensis*, inoculation with *C. etunicatum*, and co-inoculation with *B. thuringiensis* and *C. etunicatum* reduced the number of galls and emerged adults in *A. adenophora* (Figure 7e,f). Compared with the uninoculated treatment, the gall numbers of *P. utilis* in *A. adenophora* inoculated with *B. thuringiensis*, inoculated with *C. etunicatum*, and co-inoculated with *B. thuringiensis* and *C. etunicatum* decreased by 25.00%, 25.00%, and 33.33%, respectively. The emerged adults of *P. utilis* in *A. adenophora* with three inoculants were decreased by 26.32%, 10.53%, and 46.15%, respectively.

### 3.5. PCAs of Patterns of Ageratina adenophora’s Response to Inoculation with Bacillus thuringiensis or/and Claroideoglomus etunicatum and Parasitism of Procecidochares utilis

Principal components analysis for 22 plant traits of *A. adenophora* showed that the first two principal components explained 89.1% of the variance (Figure S2). The first component (PC1) represented 53.0% of the variability and accounted primarily for total chlorophyll; aboveground biomass; starch; soluble sugar; soluble protein; tannic acid; and N, P, and C concentrations. The second component (PC2) represented 36.1% of the variance and primarily comprised belowground biomass; root length; root surface area; root diameters; root volume; SA, JA, total phenol, and flavonoid concentrations; and SOD, POD, PAL, and PPO activities. Biplots from the PCA clearly showed most of plant growth indicators in the PC1 direction and most of plant defense indicators in the PC2 direction. Most of the plant growth indicators were positively related to *C. etunicatum*, *B. thuringiensis,* and *C. etunicatum* inoculation, and most of the plant defense indicators were positively related to *B. thuringiensis*, *C. etunicatum*, and *B. thuringiensis* and *C. etunicatum* inoculation.

### 3.6. Correlation of Root Colonization rate of Claroideoglomus etunicatum and the Density of Bacillus thuringiensis with Duration of Development of Procecidochares utilis

In order to explain the impact of the root colonization rate of *C. etunicatum* and density of *B. thuringiensis* on the duration of the development of *P. utilis* through affecting the growth and defense ability of *A. Adenophora*, the correlation between the colonization rate of *C. etunicatum* and the duration of the development of *P. utilis* and the correlation between the density of *B. thuringiensis* and the duration of the development of *P. utilis* were analyzed (Table S2). The root colonization rate of *C. etunicatum* was significantly positively correlated with the “galls visible” duration (*r*^2^ = 0.811, *p* = 0.03), “window” duration (*r*^2^ = 0.844, *p* = 0.026), and “adult emergence” duration (*r*^2^ = 0.762, *p* = 0.035). However, the root colonization rate was not correlated with the lifespan of adults (*r*^2^ = 0.037, *p* = 0.158), number of galls (*r*^2^ = 0.168, *p* = 0.192) or emerged adults (*r*^2^ = 0.225, *p* = 0.060). The density of *B. thuringiensis* was positively correlated with the “galls visible” duration (*r*^2^ = 0.811, *p* < 0.001), “window” duration (*r*^2^ = 0.811, *p* < 0.001), and “adult emergence” duration (*r*^2^ = 0.811, *p* < 0.001). However, the density of *B. thuringiensis* was not correlated with the lifespan of adults (*r*^2^ = 0.037, *p* = 0.158), number of galls (*r*^2^ = 0.168, *p* = 0.192) or emerged adults (*r*^2^ = 0.225, *p* = 0.060). Furthermore, regression analysis revealed that the density of *B. thuringiensis* exhibited a stronger positive correlation with the durations of the development of *P. utilis* compared to the root colonization of *C. etunicatum*, indicating that *B. thuringiensis* may more effectively inhibit the growth and development of *P. utilis* by enhancing the defensive ability of *A. Adenophora*.

## 4. Discussion

The beneficial microorganisms in the rhizosphere soil of invasive plants can create favorable environments during their invasion that promote their growth and defense through different mechanisms [62,63]. Our results showed that an AMF and *Bacillus* in the rhizosphere of *A. adenophora* play distinct roles depending on *P. utilis* parasitism. Specifically, when *A. adenophora* was not parasitized by *P. utilis*, inoculation with the AMF and dual inoculation significantly enhanced its growth. Conversely, when the plant was parasitized by *P. utilis*, inoculation with the AMF, inoculation with *Bacillus*, and dual inoculation significantly improved *A. adenophora*’s defensive ability. Consequently, *A. adenophora* can selectively accumulate bacteria and fungi with different functions [64], thereby enhancing its growth and facilitating responses to different abiotic and biotic stresses, which is advantageous for its expansion in invasive areas [15].

One primary reason for the rapid expansion of invasive plants in introduced ranges is their ability to overcome nutrient deficiencies [65]. The extraradical mycelia of AMFs can increase the nutrient absorption surface area of roots, thereby directly promoting root growth and enhancing the absorption and transfer of nutrients [66,67]. Our results showed that inoculation with *C. etunicatum* significantly improved the root growth characteristics of *A. adenophora*, resulting in increased concentrations of carbon, nitrogen, and phosphorus, which subsequently increased biomass. Parasitism by *P. utilis* significantly reduced the aboveground biomass, root growth characteristics, and nutrient concentration of *A. adenophora*. In the presence of insect parasitism, AMFs can improve plant tolerance to herbivores by enhancing plant growth, nutrient absorption, and photosynthetic rates [68,69]. Currie et al. [70] found that AMFs increased the biomass of the clover root weevil (*Sitona lepidus* Gyllenhal (Curculionidae)) feeding on white clover. Under *P. utilis* parasitism, we found that inoculation with *C. etunicatum* significantly increase the biomass and N, P, chlorophyll, sugar, and starch concentrations of *A. adenophora*, thereby enhancing its tolerance. Conversely, we observed that inoculation with *B. thuringiensis* had no significant effect on the growth and nutrient absorption of *A. adenophora* compared to the uninoculated treatment, indicating that *B. thuringiensis* does not promote growth in *A. adenophora*. Our results are consistent with the results of Fang et al. [8], which indicate that most functional bacteria in the rhizosphere of *A. adenophora* do not positively influence the plant’s performance. The growth-promoting function of bacteria on plants is influenced by plant specificity and the composition of plant root exudates [71,72]. For example, phenolic compounds secreted by the roots of the invasive plant *F. bidentis* can influence the fixation and overall availability of soil NH4+ and total nitrification, thereby affecting the growth-promoting function of *Bacillus megaterium* in the rhizosphere [73]. The absence of a growth-promoting effect of *B. thuringiensis* on *A. adenophora* may be related to the root exudates of *A. adenophora* and their impact on the soil environment, but further studies are warranted. However, under *P. utilis* parasitism, the growth and nutrition of *A. adenophora* treated with *B. thuringiensis* were significantly improved compared to those with the uninoculated treatment. This improvement may be attributed to the fact that inoculation with *B. thuringiensis* enhanced the resistance of *A. adenophora*, thereby reducing the performance of *P. utilis*. This is consistent with the results of many studies, which have found that *Bacillus* spp. enhances plant growth and yield by inducing systemic resistance in plants, thereby reducing herbivore performance [74,75].

Beneficial microorganisms, such as AMFs and *Bacillus*, can help plants to simultaneously allocate resources to both tolerance and resistance traits [68,76]. Our study showed that inoculation with *C. etunicatum* or *B. thuringiensis* significantly enhanced the activities of antioxidant defense enzymes, including PAL, PPO, POD, and SOD (Figure 4). This increase in enzyme activity not only eliminates the elevated levels of reactive oxide species (ROS) caused by insect parasitism [77] but also facilitates the production of secondary metabolites toxic to insects [78]. Balog et al. [79] found that elevated PPO and POD activity in pepper can reduce its infestation by arthropod pests. The key factors inducing resistance in the presence of AMFs and *Bacillus* are plant hormones, especially jasmonic acid (JA), which usually accumulates in plants inoculated with AMFs and *Bacillus*. Our results showed that inoculation with *C. etunicatum* or *B. thuringiensis* significantly increased the JA concentration in *A. adenophora* (Figure 5). The JA signaling pathway serves as a crucial defense mechanism in many plants, and numerous studies have shown that inoculation with AMFs and *Bacillus* can enhance plant defense capabilities by activating the JA signaling pathway [80,81]. Jiang et al. [82] found that inoculation with AMFs in *Populus alba* × *P. berolinensis* upregulated genes associated with JA synthesis and signal transduction, thereby activating the JA signaling pathway and enhancing the plant’s defense against the gypsy moth larvae. *Bacillus* spp. has been shown to induce and activate the JA signaling pathway in tomatoes, enhancing their resistance to whiteflies [83]. Our results also showed that inoculation with *C. etunicatum* or *B. thuringiensis* significantly increased the concentrations of total phenols, flavonoids, and tannins in plants (Figure 6). These secondary metabolites can inhibit feeding or digestion and exhibit other toxic activity toward herbivores, thereby inhibiting herbivores’ development [75,84]. Furthermore, our results show that AMF or *Bacillus* inoculation significantly prolonged the growth and development duration of *P. utilis*, while also reducing the number of galls and adult emergence (Figure 7 and Figure S2). We also found that inoculation with *B. thuringiensis* enhanced most of the defense indicators significantly more than did inoculation with *C. etunicatum*. However, the flavonoid concentrations of *A. adenophora* were enhanced by *C. etunicatum* significantly more than by *B. thuringiensis*. Flavonoids play an important role in AMFs’ ability to support invasive plants [85]. Future research should focus on the resistance mechanisms induced by AMFs and *Bacillus* in invasive plants, including alterations in plant morphology, the activation of the jasmonic acid signaling pathway, and the biosynthesis of defense metabolites, which will further corroborate our results.

In comparison to single inoculation, co-inoculation was more in line with the growth environment of *A. adenophora* under natural conditions, thus providing a better feedback effect. The interaction between AMFs and *Bacillus* in the rhizospheres of invasive plants has become the focus of several studies [34,35]. Our findings indicate that the AMF colonization and *Bacillus* density were significantly higher following the co-inoculation treatment than the single inoculation treatments, regardless of whether the plant was parasitized by *P. utilis* or not. Parasitism by *P. utilis* significantly reduced the colonization rate of *C. etunicatum* under the single inoculation treatment but had no significant effect under the dual inoculation treatment (Figure 1). This may be because *B. thuringiensis* and its secretions can assist in the development and colonization of *C. etunicatum* at the roots of *A. adenophora*. Botir et al. [86] also found that AMF-associated bacteria can enhance the root colonization of AMFs. The colonization rate of AMFs and density of *Bacillus* may be good indicators of plant growth [11,87]. Invasive plants, which exhibit higher AMF colonization rates and spore densities compared to native plants, are able to acquire more nutrients, thereby enhancing their growth and competitiveness. Our results demonstrate that co-inoculation treatment is more effective than single AMF inoculation in promoting the growth of *A. adenophora*, as evidenced by increases in the biomass; root growth characteristics; and concentrations of soluble sugar, protein, and starch. However, the concentrations of carbon, nitrogen, and phosphorus were not significantly different compared to AMF inoculation. The dual inoculation of *B. thuringiensis* and *C. etunicatum* resulted in greater increases in root growth characteristics and photosynthetic substance concentrations, rather than increases in other nutrient concentrations. *Bacillus* not only promotes plant growth by activating nutrients but also stimulates root growth through the secretion of auxins [88]. Our results are consistent with those of Richard et al. [89], who found that the combined action of AMFs and *Bacillus* resulted into greater root biostimulation than did single inoculation, ultimately improving the growth of *Persea americana* Mill (Lauraceae). Furthermore, compared to *B. thuringiensis* inoculation treatment, co-inoculation significantly enhanced the activities of PAL and PPO, as well as the concentrations of JA and flavonoids. This suggests that the interaction between the two microorganisms positively influences the induction of plant systemic resistance by activating the jasmonic acid pathway, which further increases enzyme activity and the concentration of secondary metabolites associated with plant resistance to herbivores [90]. The increases in these indicators resulting from the dual inoculation treatment may contribute to the extended developmental duration of *P. utilis* and inhibition of *P. utilis* growth. A limitation of our study is that we did not investigate the differences in gene expression and metabolites related to *A. adenophora* defense among the AMF, *Bacillus*, and co-inoculation treatments. Consequently, we cannot ascertain how various beneficial microorganisms induce *A. adenophora* to resist the mechanisms associated with different defenses against feeding by *P. utilis*. To enhance the effectiveness of *A. adenophora* biocontrol agents, further in-depth research into these mechanisms is essential.

Our results indicated that the dominant AMFs *C. etunicatum* and *B. thuringensis* in the rhizosphere of *A. adenophora* can enhance the defense–growth tradeoffs in this invasive species, prolonging the development duration of *P. utilis* and reducing the number of galls and emerged adults, further contributing to the spread of *A. adenophora* in invaded areas. From the perspective of beneficial microorganisms, this finding partially explains the unsatisfactory effects observed in attempts to control *A. adenophora* using the specialist herbivore *P. utilis*, allowing for the continued spread of *A. adenophora* and resulting in significant economic losses and negative effects on the ecosystem. When invasive and native plant species are closely related, invasive plants can still outcompete native plants due to herbivore feeding. Recent studies have indicated that feeding by specialist herbivores may enhance the resistance of invasive plants to these herbivores but decrease their ability to withstand abiotic stress [91]. Consequently, specialist herbivores may inhibit the invasion of these plants when they are competing with unrelated native species. Future research into the interactions among beneficial microorganisms, invasive plants, and specialized herbivores will further elucidate the mechanisms by which invasive plants could be controlled by considering competition among different native plants.

## 5. Conclusions

Our results indicate that *B. thuringiensis* aggregated in the rhizosphere of *A. adenophora* can enhance the plant’s defense ability by increasing antioxidant enzyme activity, defense hormones, and secondary metabolite concentrations, while AMFs can positively improve the nutrient content, promoting the plant’s growth and improving its defense ability. Both microorganisms can prolong the development duration of the *P. utilis* parasitizing on *A. adenophora* and inhibit its galls and emerged adults. Thus, the combination of morphological and metabolic effects obtained using these two microorganisms (AM + BT) enables *A. adenophora* to grow better and resist *P. utilis* parasitism, thereby further expanding in the invasion area. These findings highlight the need to incorporate the functions of beneficial soil microbes in future studies on the interactions between invasive plants and biological control.

## Figures and Tables

**Table 1:** Effects of different inoculant treatments and *Procecidochares utilis* parasitism on the root growth characteristics of *Ageratina adenophora.*

Inoculation Treatment	Parasitism Treatment	Root Length (m)	Root Surface Area (cm^2^)	Root Diameter (mm)	Root Volume (cm^3^)
C	No parasitism	7.97 ± 0.17 Ac	597.29 ± 15.59 Ac	0.42 ± 0.02 Ac	3.02 ± 0.27 Ac
	Parasitism	7.92 ± 0.23 Ad	584.38 ± 17.28 Ad	0.41 ± 0.02 Ad	2.93 ± 0.18 Ad
BT	No parasitism	8.11 ± 0.19 Ac	612.69 ± 13.86 Ac	0.42 ± 0.01 Ac	2.91 ± 0.23 Ac
	Parasitism	8.41 ± 0.19 Ac	654.69 ± 18.31 Ac	0.48 ± 0.02 Ac	3.72 ± 0.14 Ac
CE	No parasitism	10.33 ± 0.49 Ab	902.67 ± 14.27 Ab	0.78 ± 0.04 Ab	5.01 ± 0.32 Ab
	Parasitism	9.85 ± 0.28 Ab	887.74 ± 19.58 Ab	0.75 ± 0.04 Ab	5.03 ± 0.21 Ab
BT + CE	No parasitism	11.61 ± 0.76 Aa	955.82 ± 26.72 Aa	0.82 ± 0.02 Aa	5.49 ± 0.29 Aa
	Parasitism	11.07 ± 0.59 Aa	959.86 ± 25.60 Aa	0.80 ± 0.02 Aa	5.33 ± 0.21 Aa

C, control; BT, inoculated with *Bacillus thuringiensis*; CE, inoculated with *Claroideoglomus etunicatum*; BT + CE, inoculated with *B. thuringiensis* and *C. etunicatum*. Different lowercase letters indicate significant differences between inoculated treatment by LSD test (*p* < 0.05). Different uppercase letters indicate significant differences between parasitism by *Procecidochares utilis* and no parasitism by *t*-test (*p* < 0.05).

**Table 2:** Effects of different inoculant treatments and *Procecidochares utilis* parasitism on the nutrient composition of *Ageratina adenophora.*

Inoculation Treatment	Parasitism Treatment	Soluble Sugar (μg/mg)	Soluble Protein (μg/mg)	Starch (μg/mg)
C	No parasitism	10.41 ± 0.24 Ac	6.25 ± 0.17 Ac	8.66 ± 0.11 Ac
	Parasitism	8.09 ± 0.23 Bc	3.18 ± 0.09 Bc	5.88 ± 0.31 Bc
BT	No parasitism	10.48 ± 0.46 Ac	6.53 ± 0.26 Ac	8.36 ± 0.36 Ac
	Parasitism	9.04 ± 0.14 Bb	4.77 ± 0.14 Bb	7.41 ± 0.09 Bb
CE	No parasitism	11.42 ± 0.12 Ab	7.33 ± 0.24 Ab	9.58 ± 0.13 Ab
	Parasitism	9.14 ± 0.14 Bb	4.77 ± 0.09 Bb	7.39 ± 0.13 Bb
BT + CE	No parasitism	12.39 ± 0.29 Aa	8.81 ± 0.11 Aa	10.13 ± 0.46 Aa
	Parasitism	9.74 ± 0.12 Ba	5.18 ± 0.09 Ba	7.84 ± 0.11 Ba

C, control; BT, inoculated with *Bacillus thuringiensis*; CE, inoculated with *Claroideoglomus etunicatum*; BT + CE, inoculated with *B. thuringiensis* and *C. etunicatum*. Different lowercase letters indicate significant differences between the inoculated treatments by LSD test (*p* < 0.05). Different uppercase letters indicate significant differences between parasitism by *Procecidochares utilis* and no parasitism by *t*-test (*p* < 0.05).

## Data Availability

The original contributions presented in this study are included in the article/supplementary material. Further inquiries can be directed to the corresponding author.

## Supplementary Materials

The following supporting information can be downloaded at: https://www.mdpi.com/article/10.3390/microorganisms12122438/s1, Figure S1: Experimental design sketch; Table S1: Two-way ANOVAs of the effects of inoculation treatments and *P. utilis* parasitism on the growth indicators of *A. adenophora*; Figure S2: The principal component analysis (PCA) of plant growth indicators of *A. adenophora* under parasitim and no parasitim of *P. utilis*. The principal component analysis (PCA) of plant growth indicators of *A. adenophora* under parasitim and no parasitim of *P. utilis*. Green, control; Yellow, inoculated with *B. thuringiensis*; Blue, inoculated with *C. etunicatum*; Red, inoculated with *B. thuringiensis* and *C. etunicatum*; Table S2: Correlation among root colonization rate of *Claroideoglomus etunicatum*, density of *Bacillus thuringiensis* and duration of development of *Procecidochares utilis*.
