# Supplementary material for: Claroideoglomus etunicatum and Bacillus thuringiensis Affect the Growth of the Invasive Plant Ageratina adenophora and Its Defense Against the Specialist Herbivore Procecidochares utilis
Source: Microorganisms. 2024 Nov 27;12(12):2438. doi: 10.3390/microorganisms12122438 (PMC11676846; doi:10.3390/microorganisms12122438)
Supplement: Supplementary file 1 [file microorganisms-12-02438-s001.zip › microorganisms-3315752-supplementary.pdf]

Figure S1 Experimental design sketch

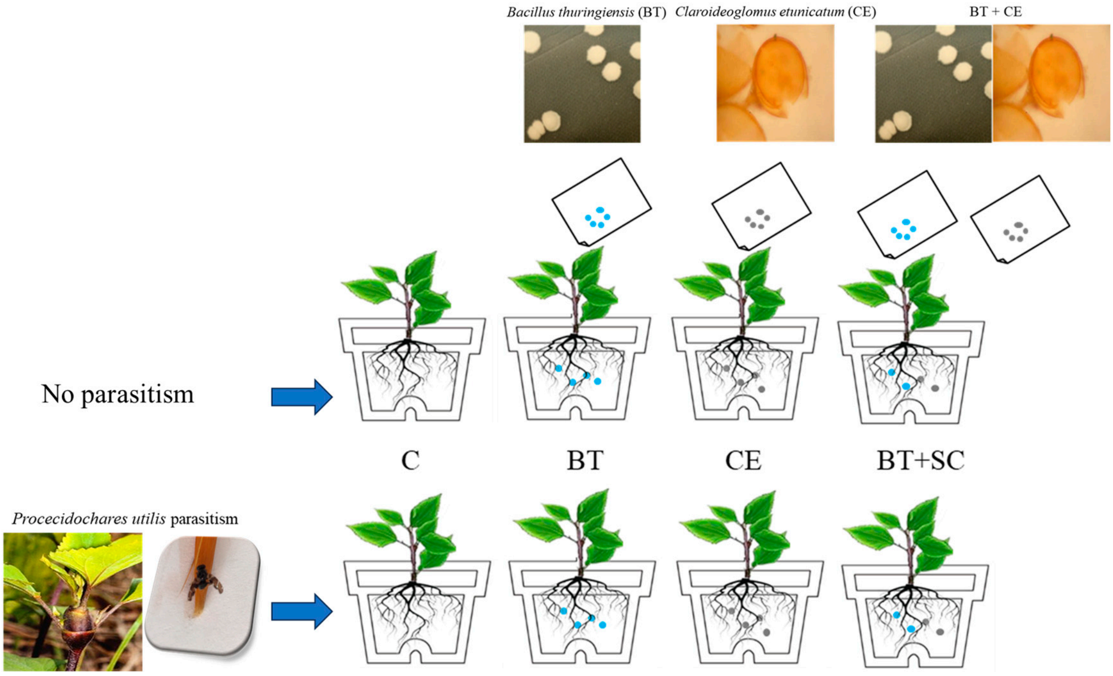

Table S1 Two-way ANOVAs of the effects of inoculation treatments and *P. utilis* parasitism on the growth indicators of *A. adenophora*

Parameters	Inoculation treatment		*P. utilis* parasitism treatment		Inoculum**P. utilis* treatment	
	F	P	F	P	F	P
Aboveground biomass	137.71	<0.001	387.31	<0.001	14.95	<0.001
Belowground biomass	400.15	<0.001	3.84	0.059	0.22	0.879
Root length	151.85	<0.001	3.28	0.08	1.47	0.242
Root surface area	1008.03	<0.001	0.83	0.37	0.52	0.675
Root diameter	769.85	<0.001	2.69	0.111	0.36	0.785
Root volume	291.46	<0.001	0.64	0.431	0.27	0.848
Soluble sugar	102.97	<0.001	805.67	<0.001	11.44	<0.001
Soluble protein	339.62	<0.001	2870.63	<0.001	59.93	<0.001
starch	86.97	<0.001	661.76	<0.001	23.85	<0.001
Chlorophyll	225.25	<0.001	868.74	<0.001	22.85	<0.001
Carbon	45.28	<0.001	1055.74	<0.001	44.14	<0.001
Nitrogen	245.49	<0.001	393.29	<0.001	35.42	<0.001
Phosphorus	137.43	<0.001	285.38	<0.001	26.04	<0.001
PAL	164.89	<0.001	1378.61	<0.001	6.93	0.001
PPO	337.82	<0.001	4226.38	<0.001	4.55	0.009
POD	329.57	<0.001	2966.51	<0.001	34.29	<0.001
SOD	782.14	<0.001	6729.91	<0.001	27.59	<0.001
JA	118.22	<0.001	580.41	<0.001	11.89	<0.001
SA	76.79	<0.001	0.85	0.364	0.505	0.682
Total phenols	457.22	<0.001	2715.19	<0.001	18.11	<0.001
Flavonoid	118.13	<0.001	1125.85	<0.001	8.73	<0.001
Tannic acid	232.09	<0.001	2600.01	<0.001	7.85	<0.001

Figure S2: The principal component analysis (PCA) of plant growth indicators of *A. adenophora* under parasitim and no parasitim of *P. utilis*. The principal component analysis (PCA) of plant growth indicators of *A. adenophora* under parasitim and no parasitim of *P. utilis*. Green, control; Yellow, inoculated with *B. thuringiensis*; Blue, inoculated with *C. etunicatum*; Red, inoculated with *B. thuringiensis* and *C. etunicatum*.

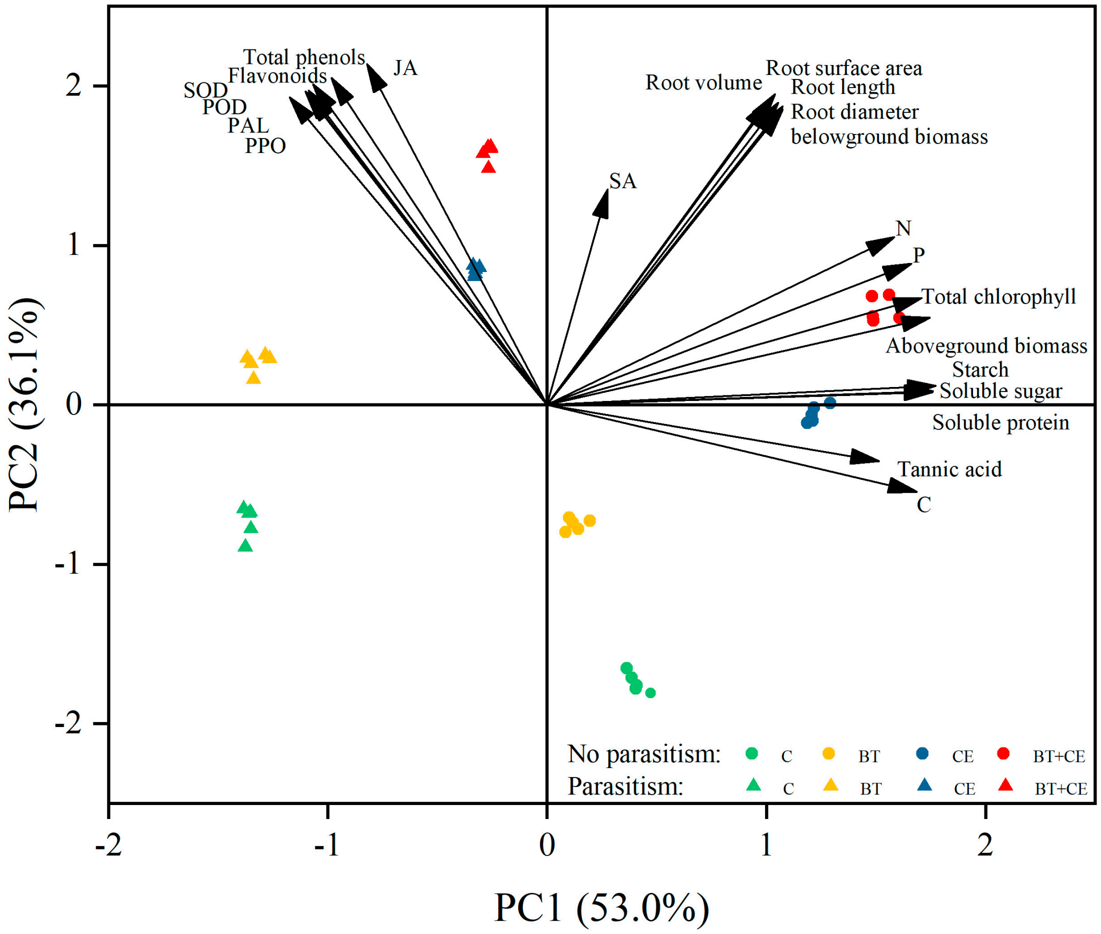

Table S2 Correlation among root colonization rate of *Claroideoglomus etunicatum*, density of *Bacillus thuringiensis* and duration of development of *Procecidochares utilis*

Indicators		Corrected R^2	B	P	VIF
Galls visible	Colonization rate of *C. etunicatum*	0.811	0.026	0.030	1.150
	Density of *B. thuringiensis*	0.811	0.062	< 0.001	1.150
Window	Colonization rate of *C. etunicatum*	0.844	0.024	0.026	1.150
	Density of *B. thuringiensis*	0.844	0.064	<0.001	1.150
Adults emergence	Colonization rate of *C. etunicatum*	0.762	0.021	0.035	1.150
	Density of *B. thuringiensis*	0.762	0.045	<0.001	1.150
Lifespan of adults	Colonization rate of *C. etunicatum*	0.037	0.002	0.873	1.150
	Density of *B. thuringiensis*	0.037	0.012	0.158	1.150
Number of galls	Colonization rate of *C. etunicatum*	0.168	-0.006	0.188	1.150
	Density of *B. thuringiensis*	0.168	-0.005	0.192	1.150
Number of emerged adults	Colonization rate of *C. etunicatum*	0.225	-0.013	0.329	1.150
	Density of *B. thuringiensis*	0.225	-0.020	0.060	1.150
